# Primary M1 macrophages as multifunctional carrier combined with PLGA nanoparticle delivering anticancer drug for efficient glioma therapy

**DOI:** 10.1080/10717544.2018.1502839

**Published:** 2018-11-22

**Authors:** Liang Pang, Ying Zhu, Jing Qin, Wenjie Zhao, Jianxin Wang

**Affiliations:** a Key Laboratory of Smart Drug Delivery, Ministry of Education & Department of Pharmaceutics, Schoolof Pharmacy, Fudan University, Shanghai, China;; b Shanghai Institute of Pharmaceutical Industry, Shanghai, China;; c Insitituteof Clinical Pharmacology, Guangzhou University of Traditional Chinese Medicine, Guangzhou, China;

**Keywords:** M1 macrophages, carrier, glioma, nanoparticle, doxorubicin

## Abstract

Glioma remains difficult to treat because of the infiltrative growth of tumor cells and their resistance to standard therapy. Despite rapid development of targeted drug delivery system, the current therapeutic efficacy is still challenging. Based on our previous studies, macrophages have been proved to be promising drug carrier for active glioma delivery. To make full use of macrophage carrier, primary M1 macrophages were proposed to replace regular macrophage to deliver nanodrugs into glioma, because M1 macrophages not only have the natural ability to home into tumor tissues, but they also have stronger phagocytic capability than other types of macrophage, which can enable them to uptake enough drug-loaded nanoparticles for therapy. In addition, M1 macrophages are not easily affected by harsh tumor microenvironment and inhibit tumor growth themselves. In this study, M1 macrophage-loaded nanoparticles (M1-NPs) were prepared by incubating poly(lactide-co-glycolide) (PLGA) nanoparticles with primary M1 macrophages. *In vitro* cell assays demonstrated M1 macrophage still maintained good tumor tropism capability after particle loading, and could efficiently carry particles across endothelial barrier into tumor tissues. *In vivo* imaging verified that M1-NPs exhibited higher brain tumor distribution than free nanoparticles. DOX@M1-NPs (doxorubicin-loaded M1-NPs) presented significantly enhanced anti-glioma effect with prolonged survival median and increased cell apoptosis. In conclusion, the results provided a new strategy exploiting M1 macrophage as carrier for drug delivery, which improved targeting efficiency and therapeutic efficacy of chemodrugs for glioma therapy.

## Introduction

Glioma, one of the refractory tumors, threatens people’s lives for rapid development and poor prognosis. It displays unique pathological characteristics from other tumors, particularly, various barriers including blood-brain barrier, blood-brain tumor barrier, and high interstitial tissue pressure rigorously prevent therapy agents from reaching the tumor sites(Juratli et al., 2013; Floyd et al., 2015; Dizon et al., 2016). In order to efficiently deliver anti-tumor drugs into the tumor, a variety of functional composited carriers were synthesized to formulate smart anti-cancer drug delivery systems (Ryu et al., 2012; Ge & Liu, 2013). However, there are still many hurdles to overcome, including complicated fabrication process, high opsonization in the circulation, low tumor targeting ability, and frequently occurred immune responses against new materials(Shiraishi et al., 2013; Verhoef et al., 2014; Peng et al., 2016).

Macrophages can be recruited into tumor tissues via chemokines such as chemokine ligand 2 (CCL2) and chemokine receptor 4 (CXCR4) (Owen & Mohamadzadeh, 2013; Jinushi & Komohara, 2015). Macrophages are prominent components of solid tumors, often comprising a major fraction of cell mass (Kelly et al., 1988; Fleige et al., 2001). Therefore, macrophages have been pursued as promising carriers of anti-cancer drugs (Chen & Liu, 2012; Anselmo & Mitragotri, 2014). Our previous studies have shown that macrophages have the ability to carry nanoparticles across the endothelial barrier into brain tissue by the mediation of cell adhesion molecules and further overcome the high interstitial fluid pressure into the core of glioma (Pang et al., 2016). Macrophages are divided into two phenotypes from oncologic viewpoint: M1 macrophages (classically activated macrophages) at one extreme and M2 macrophages (alternatively activated macrophages) at the other (Kroner et al., 2014; Pei et al., 2015). M1 macrophages can generate pro-inflammatory cytokines to help destroy foreign organisms and tumor cells (Kroner et al., 2014; Martinez & Gordon, 2014), induce T-helper-1-type cell response, and increase the production of interferon-gamma by both T and NK cells (Zhang et al., 2013; Cheema et al., 2014; Lasek et al., 2017), so M1 macrophage penetration into tumor tissues will ameliorate harsh tumor microenvironment. In addition, M1 macrophage has stronger capacity to internalize particle than regular macrophage, which can ensure therapeutic dosage (Papa et al., 2014). Moreover, the cells collected from same species can circulate for long time and avoid or decrease immunogenicity after intravenous injection into animal (Brynskikh et al., 2010). M2 macrophage in tumor tissues is often called tumor-associated macrophage (TAM). It has been proved that TAMs play positive roles in various aspects of tumor development (Qian et al., 2010; Sawawejksza et al., 2017). The existence of M2 phenotype TAMs in tumor deteriorates tumor microenvironment. Non-M2 phenotype macrophages in tumor tissues can polarize into M2 phenotype TAMs in response to harsh tumor environmental stimulus during tumor progression (Chen et al., 2011; Noy et al., 2014), whereas the conversion from mature M1 macrophage to another extreme of macrophage (M2 macrophage) requires certain stimulus and enough time (Martinez & Gordon, 2014). Considering all above, primary M1 macrophage as carrier shows superiority over regular macrophage and cell line and will not cause any burden on tumor tissues.

Poly(lactide-co-glycolide) (PLGA) is one of polymeric materials approved by the FDA, and it was generally used in drug delivery systems due to controlled and sustained-release properties, low toxicity, and biocompatibility. In our studies, it was found the DOX-loaded PLGA nanoparticles (DOX@NPs) instead of DOX was loaded into M1 macrophage, which delayed the toxicity of DOX to M1 macrophage (Pang et al., 2016). Hence, the combinational utilization of M1 macrophage and PLGA nanoparticle would be more effective drug delivery system for tumor therapy.

Concerning all the above explorations, we hypothesized that M1 incorporated DOX@NPs (M1-NPs) enabled therapeutic loading without impacting the tumor homing property of cell carriers. It would maintain good stability and be capable of actively accumulating in tumor after intravenous injection. The released DOX and M1 macrophage would induce cell apoptosis together, resulting in effective inhibition of tumor growth.

In this study, M1-NPs were produced by loading DOX@NPs into bone marrow-derived M1 macrophage. The tumor targeting ability and therapeutic efficacy of M1-NPs were compared with those of naked nanoparticles. The results showed that primary M1 macrophages could lead to favorable cellular interaction with tumor cells and facilitate the penetration of NPs into tumor tissue in U87 glioma model.

## Materials and methods

## Materials

The PLGA (LA: GA =75:25, MW: 12,000 Da) was kindly provided by Evonik (Hanau, Germany). Emprove exp poly(vinyl alcohol) (PVA) 4–88 was given as a present from Merck (Darmstadt, Germany). Doxorubicin hydrochloride (DOX·HCL) and daunorubicin were obtained from Melonapharma (Dalian, China). Interferon-γ (IFN-γ), tumor necrosis factor (TNF-α), and macrophage colony-stimulating factor (MCSF) were purchased from Peprotech (Rocky Hill, NJ). Lipopolysaccharide (LPS) was provided from Sigma (St. Louis, MO). 1,1′-Dioctadecyl-3,3,3′,3′-tetramethyl indotricarbocyanine iodide (DiR) and 1,1-dioctadecyl-3,3,3,3-tetramethylindodicarbocyanine (DiD) were purchased from Caliper (Newton, MA) and Thermo Fisher (Waltham, MA), respectively. Coumarin 6 was obtained from Aladdin (Shanghai, China). 4,6-Diamidino-2-phenylindole (DAPI) was from Beyotime (Haimen, China). All antibodies and fixation and permeabilization buffer were purchased from eBioscience (Grand Island, NY).

U87 cells and human umbilical vascular endothelial cells (HUVECs) were obtained from Shanghai Institute of Cell Biology. All cell culture reagents were purchased from Corning, Inc. (Christiansburg, VA) except Gibco fetal bovine serum.

BALB/c nude mice (female, 4–6 weeks, 20–22 g) were obtained from Shanghai B&K Lab Animal Ltd and Shanghai SLAC laboratory animal Co. Ltd (Shanghai, China), respectively, and housed under SPF conditions with free access to food and water. The animal experiment protocol was approved by the Animal Experimentation Ethics Committee of Fudan University.

### Preparation of M1 macrophages and phenotype identification

Bone marrow cells were collected by flushing the tibias and femurs of BALB/c female mice 4–6 weeks of age and then cultured for 7 days in Dulbecco’s modified Eagle medium (DMEM) supplemented with 20 ng/mL MCSF. 500 ng/mL LPS and 20 ng/mL IFN-γ in fresh DMEM medium were added and incubated for extra 48 h for cell differentiation. They were finally washed three times with PBS and cultured in fresh DMEM until use.

1 × 10^6^ cells were, respectively, incubated with antibody (anti-CD16/32 PE, anti-CD86 FITC) in 200 µL cell staining buffer for 30 min at 4 °C. Meanwhile, the same number of cells was sequentially fixed in 100 µL fixation buffer, permeabilized and incubated with anti-iNOS APC in permeabilization buffer for 30 min at 4 °C. All the cell samples were washed with PBS twice via centrifugation and tested by fluorescence-activated cell sorter (FACS) at appropriate wavelength.

### Distribution of M1 macrophages in orthotopic glioma model mouse

The DiD-labeled M1 macrophages were acquired by incubating M1 macrophages with DiD dye for 0.5 h. The intracranial U87 glioma-bearing mice model was established as previously described (Pang et al., 2016). Two weeks after inoculation, the labeled M1 macrophages were intravenously injected into mouse model. The distribution of M1 macrophages was tracked by *in vivo* imaging (IVIS Spectrum, Caliper, Newton, MA) at 644/665 nm (excitation/emission) wavelength.

### Preparation and characterization of different formulations

#### Preparation of DOX-loaded nanoparticles (DOX@NPs)

The DOX@NPs were prepared using emulsion solvent evaporation method (Tewes et al., 2007). Briefly, 20 mg PLGA was dissolved in ethyl acetate, and 100 µL of 10 mg/mL DOX·HCL solution in PBS was followingly added into it. The single emulsion was formed after sonication, to which 2 ml of 2% PVA was added immediately followed by sonication in ice bath to finally form double emulsion. It was dispersed into 2% PVA solution, stirred for 2 h, and evaporated for 30 min. The particles were acquired and washed with by centrifugation. Due to DiR and coumarin 6 are water insoluble , their particles preparation was referred to our previously reported procedures (Pang et al., 2016), which was a little different from that of DOX@NPs. Particle size, polydispersity index, and zeta potential were measured using a dynamic light scattering detector (Zetasizer, Nano ZS, Malvern, UK). The drug loading capacity of DOX@NPs was calculated as follows:

Loading efficiency(%)=(the drug feeding amount-the drug amount in supernatant)/weight of lyophilized nanoparticles×100%

#### Preparation of M1 incorporated DOX@NPs (DOX@M1-NPs)

DOX@M1-NPs were prepared by incubating M1 macrophages with DOX@NPs in FBS-free DMEM. The cells were cultured in 24-well plates at 30,000 cells per mL and incubated with 5, 10, 20, and 30 µg/mL (DOX-equivalent) of nanoparticles at 37 °C for 4, 6, 8, and 10 h. At each time point, the cells were collected and washed twice with PBS and tested using FACS. The toxicity of DOX-NPs with different concentration (5, 10, 20, 30, and 45 µg/mL, DOX equivalent) on M1 macrophage was tested using MTT assay.

In order to compare the uptake differences between M0 and M1 macrophages, they were seeded, respectively, using the same conditions as above and treated with 30 µg/mL DOX@NPs for 8 h. The free or loosely bound nanoparticles were removed by washing with PBS twice. Finally, the fluorescence intensity of the collected cells was quantified with FACS.

#### Drug loading of DOX@M1-NPs

The total DOX amount loaded in M1 macrophages was determined with HPLC (Shimadzu 1200, Kyoto, Japan). After the M1-NPs were counted and ultrasonicated, 200 µL cell lysate was added with 20 µL of 20 mg/mL daunorubicin (DAU) as internal standard and subsequently extracted with 2 mL chloroform/methanol (4:1, v/v) by vortex mixing for 2 min. After centrifuged for 5 min at 10,000 rpm, the organic phase of the mixture was collected and evaporated under nitrogen blow. The residues were then dissolved in 100 µL of mobile phase and centrifugated to collect supernatant for HPLC analysis. The concentration of the drug was assayed using C18 column (Agilent) at 230 nm wavelength over the concentration range of 0.5–10 µg/mL. The mobile phase was composed of 0.01 M KH_2_PO4, methanol, and acetic acid (40:60:0.4, v/v/v).

#### Morphology of DOX@NPs and DOX@M1-NPs

The morphology of DOX@NPs and M1-NPs was examined with transmission electron microscope (Tecnai G2-20, FEI). DOX@NPs were stained with 1% phosphotungstic acid and observed. DOX@M1-NPs underwent pre- and post-fixation and then were gradiently dehydrated in orderly ascending concentration of alcohol and embedded in an epoxy-type resin. The samples were then sectioned, contrasted with heavy metal, and observed.

### *In vitro* DOX release

DOX@M1-NPs were suspended in ultra-low attachment well plates (Corning, Christiansburg, VA) with fresh 10% FBS containing DMEM in 5 × 10^5^/mL/well. The cell samples were collected and centrifuged at each time point (0, 4, 8, 12, 24, and 48 h). Each time point has independent three samples. The DOX amount in cell pellets and supernatant were, respectively, determined with HPLC according to the method in section ‘Drug loading of DOX@M1-NPs’. To visualize drug release from M1-NPs, the DOX@M1-NPs were cultured in glass cell dishes and observed under confocal microscopy at 0, 24 and 48 h.

### *In vitro* migration

The tumor tropic ability of M1 or M1-NPs toward U87 tumor cells *in vitro* was examined by transwell migration test (Sadhukha et al., 2014; Fu et al., 2015). 10^5^ M1 macrophages loaded with DOX@NPs or not were suspended in 200 µL serum-free medium and plated in the upper chamber of the transwell. The lower compartment was filled with 600 µL of conditioned serum-free medium, which was collected from serum-free medium after culturing U87 cells for 48 h. After incubated at 37 °C for 6 h, the cells remained at the upper membrane surface were removed by cotton swab. For cell visualization, the cells migrated to the lower membrane surface were fixed in 4% paraformaldehyde and washed, stained with DAPI (1 mg/mL) for 10 min and sufficiently washed again, and then observed using fluorescence inverted microscope (Leica DMI4000D, Wetzlar, Germany). The pictures were captured with 10× objective lens. For the quantitative comparison of cell migration, the cells were detached from the transwell membrane, sonicated and detected protein amount using BCA Protein Assay Kit (Thermo Fisher, Waltham, MA).

### *In vitro* transport across endothelium

Human umbilical vein endothelial cells (HUVECs) were plated in the Millicell^®^ insert (8 µm pore size) and U87 cells were seeded into the bottom chamber. Transendothelial electrical resistance (TEER) was detected with an epithelial volt-Ωm (Millicell ERS, Millipore, Burlington, MA) to evaluate the integrity of cell monolayer. HUVEC monolayers with TEER over 300 Ω·cm^2^ were used for further experiments. 20 ng/mL TNF-α was followingly added and incubated for 24 h to induce the overexpression of cell adhesion molecules (E-selectin, VCAM-1, ICAM-1, and PECAM-1) in HUVEC monolayer (Jaczewska et al., 2014). The culture medium in each apical chamber was then replaced by coumarin 6-loaded NPs or M1-NPs in DMEM with 10% FBS, respectively. U87 cells were collected after 12-h incubation, washed and detected the fluorescence intensity by FACS.

### *In vivo* imaging

*In vivo* imaging was performed in intracranial glioma-bearing mice. Two weeks later, after inoculation, DiR-loaded NPs and M1-NPs were prepared and intravenously administrated to tumor-bearing mouse, respectively. At predetermined time points, the whole-body fluorescence distribution was acquired using *in vivo* imaging system (IVIS spectrum, Caliper, Newton, MA), and the main organs were dissected for *ex vivo* imaging 24 h post-injection.

### *In vitro* cytotoxicity

Cytotoxicity potential of different DOX dosage was determined in U87 cells. U87 cells were seeded into 96-well plate at a density of 3 × 10^3^ cells/200 µL per well. After 24-h cultivation at 37 °C, the cells were treated with different concentration of DOX, DOX@NPs, and DOX@M1-NPs. Exceptionally, the direct M1-NPs treatment will interfere the tested results due to the mixture of M1 and U87, different density of M1-NPs was added on the top donor wells of a Transwell^®^ 96-well plate, with the adherent tumor cells seeded in the bottom chamber. The cytotoxicity was determined after 48-h incubation by MTT assay.

### *In vivo* anti-tumor efficacy

#### Median survival

*In vivo* anti-tumor efficacy was performed in intracranial glioma-bearing mice (Pang et al., 2016). The mice were randomly divided into five groups (*n* = 6) and administered with saline, DOX, DOX@NPs, M1, and DOX@M1-NPs at a dose of 2 mg/kg DOX at 9, 12, 15, and 18 days after inoculation, respectively. Survival time was recorded every day and Kaplan–Meier survival curves were plotted for each group.

#### Caspase-3 expression

Fifteen intracranial glioma-bearing mice were divided into five groups randomly. Two days after the last administration, three mice in each group were anesthetized and perfused from heart with saline followed 4% paraformaldehyde. The brains were collected, fixed with 4% paraformaldehyde overnight, paraffin embedded, and sliced into 5-µm thick sections. The brain sections were stained with anti-caspase-3 antibody and visualized under fluorescence microscopy.

#### Safety evaluation

The preparations of main organ sections were simultaneously operated similarly with that described in the section ‘Caspase-3 expression’. They were stained using hematoxylin and eosin (H&E) to evaluate the safety of different formulations.

### Statistical analysis

All statistical analysis was performed with GraphPad Prism 7. Data were analyzed with one-way ANOVA test or Student’s *t*-test. A value of *p* < .05 was considered statistically significant.

## Results

### Macrophage phenotype and its tumor tropic capacity

**Figure 1.**
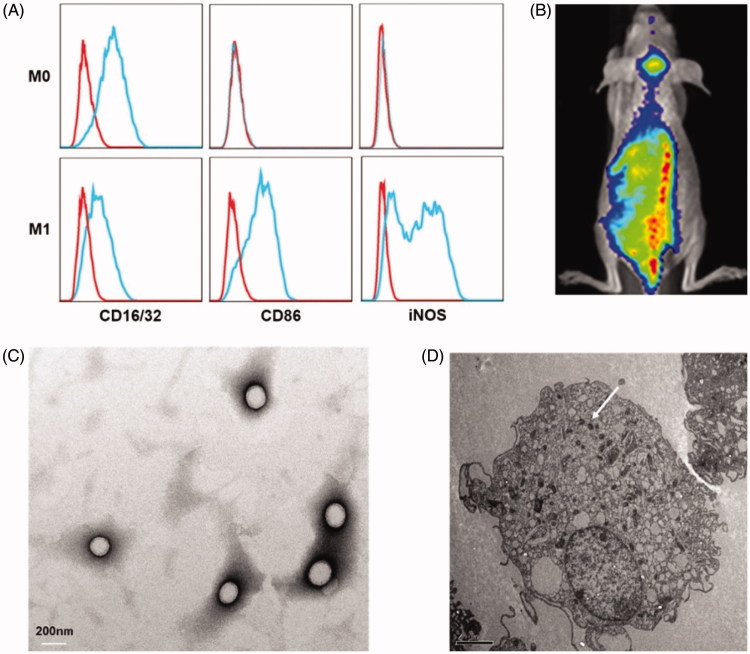
(A) M1 macrophages phenotype. Both M0 and M1 stain positively for CD16/32. M1 macrophages specifically overexpressed CD86 and iNOS. (B) The distribution of M1 macrophages in orthotopic glioma mouse 12 h after intravenous injection. Transmission electronic microscopy of DOX@NPs (C) and M1 macrophage-loaded NPs (white arrow indicated NP) (D).

The biomarkers of different macrophages were tested using FACS, which can identify the phenotype of the cells after different cytokines treatment. As shown in Figure 1(A), the cells with only MCSF incubation overexpressed CD16/32, which is generally expressed on all kinds of macrophages, and therefore these macrophages were defined as M0 macrophage. The cells additionally treated with LPS and IFN-γ specifically expressed CD86 and iNOS, which were considered as M1 macrophage biomarker (Martinez & Gordon, 2014). Since DiD dye can firmly insert into phospholipid bilayer membrane by hydrophobic interaction, the biodistribution of M1 macrophages can be reflected by tracing DiD signal. *In vivo* imaging displayed a large number of DiD-labeled macrophages accumulated in the brain tumor area 12 hours after injection (Figure 1(B)), indicating the natural tumor-tropic property of M1 macrophages.

### Characterization of DOX@NPs and DOX@M1-NPs

**Figure 2.**
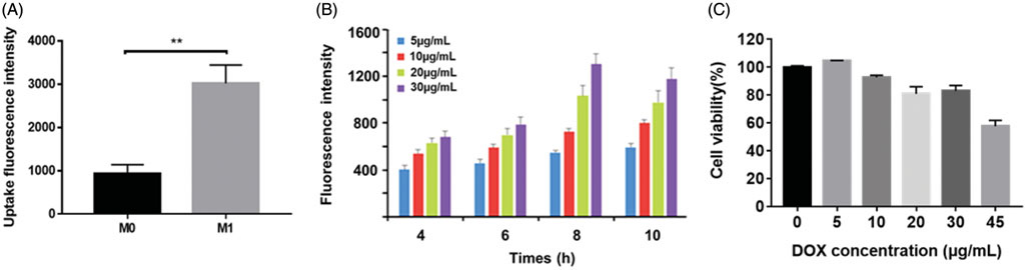
(A) The comparison of uptake differences between M0 and M1. **p < .01 by Student’s *t*-test. (B) The effect of particle concentration and incubation time on M1 macrophages uptake. (C) Cytotoxic effect of different concentration DOX@NPs on M1 macrophages after 8 h of exposure (*n* = 3).

DOX@NPs were observed to be spherical nanoparticles (Figure 1(C)) with average size of 156.9 ± 7.1 nm and the drug loading (%) was 4.35%±0.56. M1-NPs were developed by simply incubating M1 macrophages with DOX@NPs. The presence of DOX in cytoplasm of M1 macrophages was identified by TEM (Figure 1(D)). By comparison, the nanoparticle amount uptake by M1 macrophages was significantly higher than that of M0 macrophage (Figure 2(A)). It is likely due to the fact that activated M1 macrophages have stronger capacity to phagocytize more particles than unactivated M0 macrophage. Therefore, M1 macrophages were chosen as effective drug vehicle with high payload for targeting delivery. Although the cellular uptake of nanoparticles on M1 macrophages increased in concentration- and time-dependent manner in eight hours, longer incubation time over eight hours caused lower cell uptake (Figure 2(B)). DOX@NPs did not show significant toxicity to M1 macrophages after 8-h incubation when the concentration reached 30 µg/mL (Figure 2(C)). However, when the concentration increased to 45 µg/mL, the cell viability was decreased obviously to about 60%. Therefore, M1 treated with nanoparticle dose of 30 µg/mL for 8 h was used in future studies. With this incubation condition, the content of DOX loaded in M1 macrophages was determined to be 34.0 ± 2.3 μg/5 × 10^6^ cells.

### Release kinetics of DOX from M1-NPs

**Figure 3.**
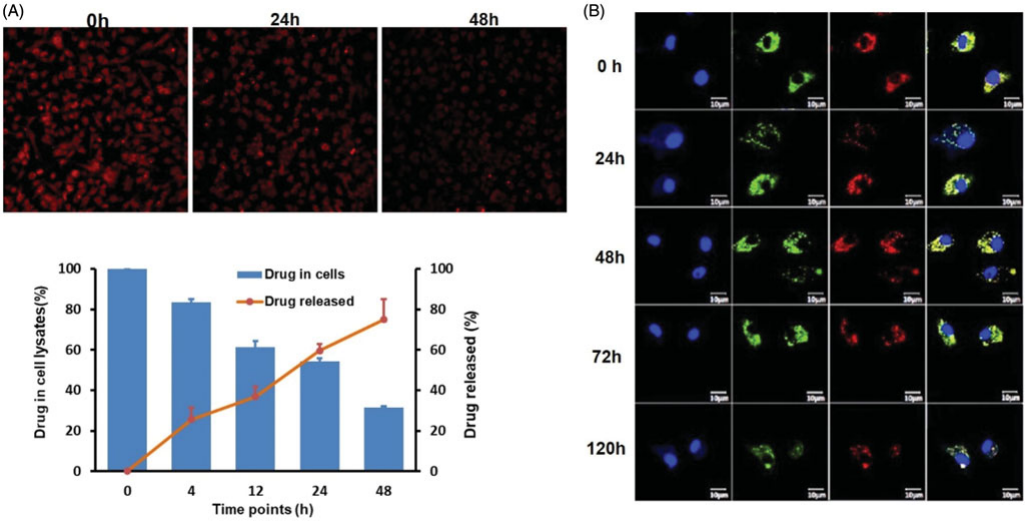
*In vivo* release profile of DOX from DOX@M1-NPs. (A) Visual observation of DOX@M1-NPs under fluorescent microscopy at 0, 24, and 48 h. (B) Quantification of DOX released from M1 macrophages and remained in them. (C) Distribution of DOX@NPs in M1 macrophages at different time points. Blue color showed cell nuclear staining with DAPI; green color represented lysosome, and red color indicated DOX, while yellow color stood for the colocalization of lysosome and DOX.

The release of DOX or DOX@NPs from M1 macrophages was studied to elucidate the stability of M1-NPs in 10% FBS containing DMEM. Persistent drug release could be visualized under fluorescence microscopy at three different time points (Figure 3(A)). The drug release and retention in M1 macrophages were, respectively, quantified using HPLC. The drug amount released into medium and remained in M1 macrophages roughly reached 100%. Less than 40% drug was released in first 12 h with 65% retention in the cells, and about 73% of the drug was released in 48 h with 26% leftover in M1 macrophages (Figure 3(B)). In conclusion, the DOX@M1-NPs keep relatively stable in 10% FBS containing DMEM, and the drug was sustainably released from it in slowly way.

The behavior of DOX@NPs in M1 macrophages was observed by labeling under confocal imaging. The particle signal always existed in cytoplasm and colocalized with lysosome at all the predetermined time points, and no DOX signal was detected in cell nucleus (Figure 3(C)). The result indicated DOX@NPs mainly located in lysosome after loading into M1 macrophages. It was supposed that it will be gradually released by exocytosis in exosome form.

### The chemotaxis of M1-NPs toward U87 conditioned medium

**Figure 4.**
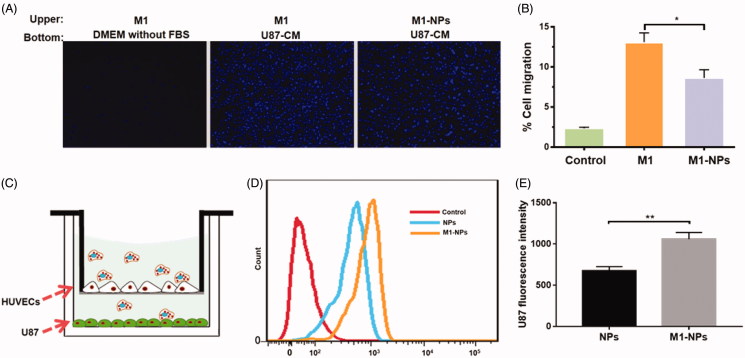
(A) Demonstration of M1 or M1-NPs migrates through transwell membrane pore toward U87 condition medium (left: negative control, M1 + DMEM without FBS; middle: M1 + U87-CM; right: M1-NPs + U87-CM). (B) Quantification of the migrated M1 or M1-NPs (*n* = 5). *p < .05 by one-way ANOVA test. (C) Schematic illustration of *in vitro* endothelial barrier model. (D,E) U87 fluorescence intensity was tested by FACS (*n* = 3). **p < .01 by Student’s *t*-test.

The migration of free M1 and M1-NPs toward U87 conditioned medium was compared using a 24-well transwell system. As shown in Figure 4(A,B), free M1 macrophages migration toward the fresh serum-free DMEM was set as control. It was demonstrated that minimal migration was observed in control group due to the lack of chemokines in fresh DMEM. 13% of the M1 macrophages migrated to the bottom compartment in 6 h when U87 conditioned medium (U87-CM) was applied in the bottom chamber. In contrast, only 8% of the M1-NPs moved across the transwell membrane. Although particle loading statistically slowed down the migration of M1 macrophages toward tumor, it was clear that the tumor homing capacity of M1-NPs was still promising.

### Transmigration of M1-NPs across the endothelial barrier model

The *in vitro* endothelial barrier model was established to assess the transcytosis efficiency of particles under tumor microenvironment (Figure 4(C)) (Penberthy et al., 1997; Wong et al., 2007; Parodi et al., 2013). The TEER gradually increased and reached plateau around 12–14 days (around 500Ω·cm^2^). The addition of TNF-α induced the overexpression of cell adhesion molecules in HUVEC monolayer, which can better mimic the endothelial barrier under tumor environment (Jaczewska et al., 2014). NPs and M1-NPs were added into upper chamber, respectively, and incubated for 12 h. As shown in Figure 4(D,E), the U87 uptake fluorescence intensity in M1-NPs treatment is 1.6-fold higher than single NPs treatment. Therefore, M1 as carrier significantly boosted the transcytosis of nanoparticles across the endothelial barrier and uptake by U87 in the bottom.

### *In vivo* tumor targeting

**Figure 5.**
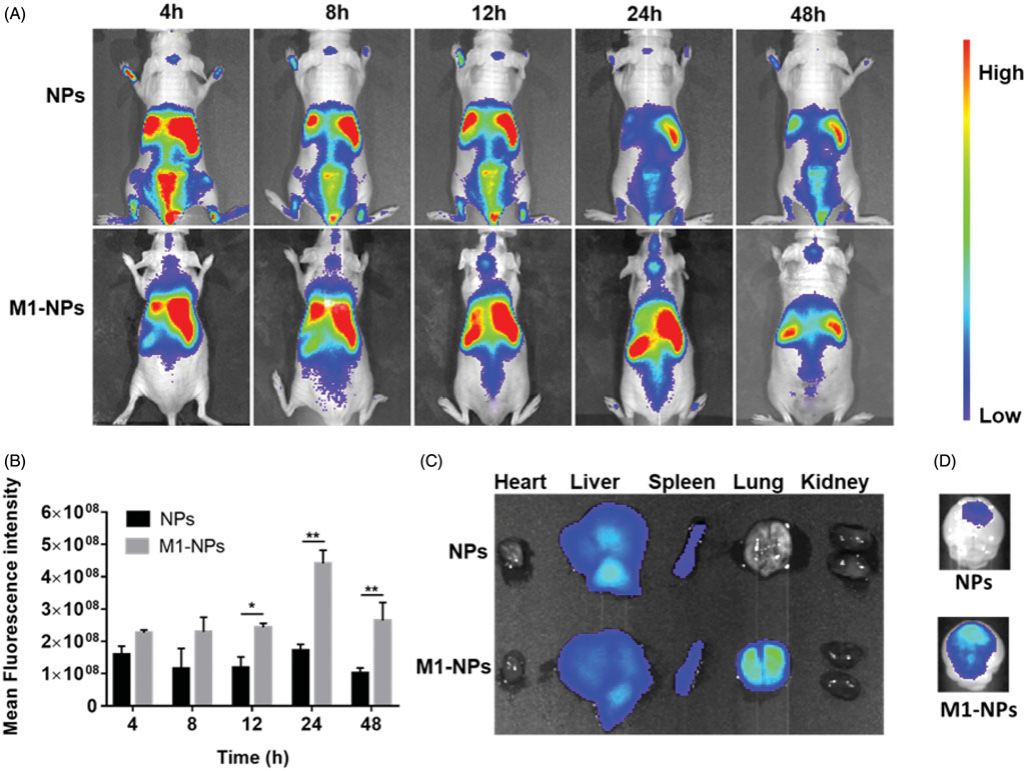
The whole-body imaging after tail vein injection with DiR loading NPs and M1-NPs (A). Semi-quantitative ROI analysis of the average fluorescence intensity in brain from the NPs and M1-NPs group (mean ± SD, *n* = 3); *p < .05, **p < .01 by Student’s t-test (B). *Ex vivo* imaging of main organs (C) and brains (D) 24 h post-injection.

*In vivo* imaging experiments were performed to evaluate the distribution of NPs and M1-NPs in nude mice bearing intracranial U87 glioma. The accumulation of NPs in tumors was limited, while M1-NPs exhibited significant superiority in glioma targeting with high fluorescence intensity at all time points (Figure 5(A,B)). The distribution of NPs and M1-NPs in main organs was different: NPs mainly distributed in liver and spleen, while M1-NPs entered into liver, spleen, and lung. This is likely attributed to the size differences between NPs and M1-NPs. M1-NPs in around 10 µm diameter easily stuck in blood capillary enriched lung (Figure 5(C)). *Ex vivo* brain imaging showed that weak fluorescence was observed in the glioma of mice treated with free nanoparticles, but significantly greater distribution of nanoparticle in the glioma was detected when treated with M1-NPs (Figure 5(D)).

### *In vitro* cytotoxicity

The cytotoxic potential of different DOX formulations was determined in U87 cells. IC50 of free DOX- and DOX-loaded nanoparticles was 0.695 and 0.639 µg/mL, respectively, while M1-NPs displayed the strongest cytotoxicity among the groups, whose IC50 value was only 0.311 µg/mL. This is likely due to the tremendously enhanced cellular uptake. It is speculated that DOX@NPs can be secreted from M1 in exosome form by exocytosis, which can easily fuse with tumor cell membrane and enter into it (Zhang et al., 2017).

### *In vivo* efficacy and safety evaluation

**Figure 6.**
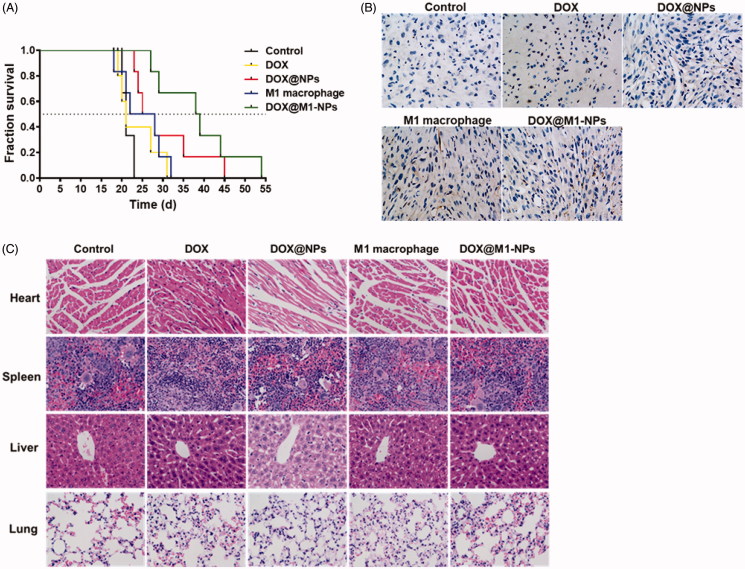
*In vivo* anti-cancer effect after five different formulations on orthotopic U87 glioma-bearing mouse. (A) Kaplan–Meier analysis of survival (*n* = 6). (B) The expression of caspase-3 protein in brain tumor. Nucleus was labeled DAPI (blue); caspase-3 was stained by anti-caspase-3 antibody (brown). (C) H&E sections of main organs from U87 glioma-bearing mice.

The anti-glioma effect was performed on orthotopic U87 glioma-bearing mice model and survival evaluation was presented in a Kaplan–Meier plot. As depicted in Figure 6(A), DOX@NPs, M1 treatment group all elicited slight extension in life span over DOX and PBS control group, with their median orderly being 26.5 days, 25 days, 21 days, and 21 days, respectively. It was noteworthy that M1-NPs significantly prolonged mice survival with median survival 38.5 days (M1-NPs vs. all other groups, *p* < .05).

Cell apoptosis of tumor tissues was evaluated based on caspase-3 protein expression. Caspase-3, a frequently activated death protease, is crucial mediator of programmed cell death (apoptosis), catalyzing the specific cleavage of many key cellular proteins (Kobayashi et al., 2007). Among all the treatment groups, mice received DOX@M1-NPs displayed the highest caspase-3 expression, traces of caspase-3 expression were found in M1 macrophages and DOX@NPs group, and no apoptosis signal was detected in control and DOX groups (Figure 6(B)). These results indicated that DOX@M1-NPs exhibited a significant improvement in anti-tumor activities.

It was known that DOX has serious adverse effects, especially lethal cardiotoxicity. To further evaluate whether these formulations injured heart and their distributed organs, the tissue sections were H&E-stained. As shown in Figure 6(C), myocardial hypertrophy was predominately observed in free DOX treatment, and there was no injury found in other organs among all these formulations including M1-NPs.

## Discussion

Cell-mediated drug delivery system offers several advantages over other systems, which include long circulation, specific tropism to diseased tissues, sustained drug release, and limited immunogenicity (Anselmo & Mitragotri, 2014; Pang et al., 2016). The early attempt was to synthesize ligand-modified liposomes, which were captured by neutrophils in circulation after injection; then, the liposomes were indirectly transported into the site of lesion in stroke model (Qin et al., 2007; Zhang et al., 2017). Later, biomimetic drug system was constructed by directly loading DOX into mouse macrophage cell line RAW264.7. It was certified that it was a potential drug delivery system for targeted cancer therapy. However, the P-glycoprotein expressed in RAW264 provided the driving force to pump drug molecule outside, which caused drug release during circulation in advance. In addition, the direct contact between DOX and macrophage increased cell volume due to cytotoxicity, which further compromised the tumor tropism capability (Fu et al., 2015). It was hypothesized that using nanoparticle instead of free drug can overcome these problems. On the one hand, it would avoid the efflux effect of P-glycoprotein; on the other hand, it can delay the toxicity of drug to cell carrier by blocking the direct contact between cell carrier and drug. Therefore, DOX-loaded PLGA nanoparticles were adopted to load into M1 macrophage in our studies. In order to make the best use of cell as carrier, it was expected that the cell carrier would be conferred more functions. M1 macrophages itself were reported to suppress tumor growth and stimulate body immune response via secreted inflammatory cytokines, such as IL-12 and TNF-α (Solinas et al., 2009; Martinez & Gordon, 2014). Hence, M1 macrophage as carrier not only delivered drug into tumor sites, but also inhibit tumor growth. Furthermore, it was found in our studies the phagocytic capability of M0 macrophage was so limited that it is difficult to meet therapy needs, while M1 macrophages as activated macrophage can uptake particles threefold higher than that of M0 macrophages, which can satisfy therapeutic dosage.

In our studies, primary M1 macrophages were acquired by collecting bone marrow cells and cultivating in conditioned medium for several days. DOX@NPs were loaded into M1 macrophages by *in vitro* incubation, and optimized M1-NPs based on cellular uptake evaluation were determined aimed at achieving the maximum particle loading. The DOX@NPs in M1 macrophage were primarily visualized in lysosome without particle signal in nucleus, while free DOX entered into nucleus after incubation with macrophage. Thus, particle loading protected M1 macrophage from damage. The DOX in M1 macrophages was gradually and slowly released in 10% FBS containing DMEM. The number of M1-NPs moving toward U87 conditional medium in the bottom chamber decreased a little compared with free M1 macrophages in first 6 hours, and it indicated that more payload in macrophage would slow down motor ability of macrophage, which makes the behavior of tumor chemotaxis lag behind single M1 cell. This is likely due to the decreased flexibility of M1 macrophages after particle loading, which renders the cell to not pass through membrane pores easily. Despite that, the tumor homing capacity still maintained promising tumor tropism capability. The biggest barrier limited drug delivery for glioma treatment is how to pass through endothelial barrier into tumor (Barua & Mitragotri, 2014; Kreuter 2014). When tumor occurs, the cell adhesion molecules on the vascular endothelial cells are overexpressed due to inflammation environment, which facilitate the initial process of macrophage rolling, firm attachment to endothelium, and transmigration (Sutton et al., 2014; Vestweber 2015). DOX@M1-NPs could efficiently transverse endothelial barrier and infiltrate into glioma via cell adhesion molecule interaction, while limited DOX@NPs were delivered into tumor tissue due to blood-brain barrier, blood–tumor barrier, and high interstitial pressure in tumor. Hence, M1 as carrier could overcome these barriers and result in a precise and high glioma retention. The drug release kinetics of M1-NPs decided their *in vivo* efficacy once it infiltrated into tumor tissues, and tumor inflammation environment accelerated drug release by exocytosis (Klyachko et al., 2014; Pang et al., 2016). Besides, the direct contact between M1 and target cell also facilitates drug transfer from macrophage into target tumor cell (Haney et al., 2012; Tao et al., 2013). *In vivo* anti-tumor efficiency and safety evaluation were implemented in mice bearing intracranial U87 glioma cells. Compared to all other groups, M1-NPs exhibited longer survival time, highest caspase-3 expression. M1-NPs reduced the cardiotoxicity of DOX and did not cause damage to their distributed organs. Taking these together, M1-NPs could serve as an efficient and safe formulation for glioma therapy.

## Conclusions

We here established and optimized drug delivery system mediated by M1 macrophages to deliver DOX@NPs for glioma therapy. This system remained relatively stable in FBS containing medium and sustained released DOX. It was demonstrated that the ability of migration and infiltration of M1-NPs into tumor tissues were efficient. Furthermore, it displayed good *in vivo* anti-tumor activity with prolonged survival time and increased caspase-3 protein expression. These promising facts suggested M1-NPs provide a valuable candidate for various anti-cancer agents in fields of drug delivery system.
